# Saffron with resistance exercise improves diabetic parameters through the GLUT4/AMPK pathway *in-vitro* and *in-vivo*

**DOI:** 10.1038/srep25139

**Published:** 2016-04-28

**Authors:** Firouzeh Dehghan, Fatemeh Hajiaghaalipour, Ashril Yusof, Sekaran Muniandy, Seyed Ali Hosseini, Sedigheh Heydari, Landa Zeenelabdin Ali Salim, Mohammad Ali Azarbayjani

**Affiliations:** 1 Department of Exercise Science, Sports Centre, University of Malaya, 50603 Kuala lumpur, Malaysia; 2 Department of Molecular Medicine, Faculty of Medicine, University of Malaya, 50603 Kuala Lumpur, Malaysia; 3 Department of Physical Education and Sport Science, Marvdasht Branch, Islamic Azad University, Marvdasht, Iran; 4 Department of Pharmacy, Faculty of Medicine, University of Malaya, Kuala Lumpur, Malaysia; 5 Department of Exercise Physiology, Central Tehran Branch, Islamic Azad University, Tehran, Iran

## Abstract

Saffron is consumed as food and medicine to treat several illnesses. This study elucidates the saffron effectiveness on diabetic parameters *in-vitro* and combined with resistance exercise *in-vivo*. The antioxidant properties of saffron was examined. Insulin secretion and glucose uptake were examined by cultured RIN-5F and L6 myotubes cells. The expressions of GLUT2, GLUT4, and AMPKα were determined by Western blot. Diabetic and non-diabetic male rats were divided into: control, training, extract treatment, training + extract treatment and metformin. The exercise and 40 mg/kg/day saffron treatments were carried out for six weeks. The antioxidant capacity of saffron was higher compare to positive control (P < 0.01). High dose of saffron stimulated insulin release in RIN-5F cells and improved glucose uptake in L6 myotubes. GLUT4 and AMPKα expressions increased in both doses of saffron (P < 0.01), whereas GLUT2 not changed (p > 0.05). Serum glucose, cholesterol, triglyceride, low-density lipoprotein, very low-density lipoprotein, insulin resistance, and glycated hemoglobin levels decreased in treated rats compared to untreated (p < 0.01). However, no significant differences were observed in the high-density lipoprotein, insulin, adiponectin, and leptin concentration levels in all groups (p > 0.05). The findings suggest that saffron consuming alongside exercise could improve diabetic parameters through redox-mediated mechanisms and GLUT4/AMPK pathway to entrap glucose uptake.

Diabetes mellitus is a metabolic disorder characterized by high blood glucose level resulting from insufficient insulin production and/or action^1^. Diabetes is characterized by increased level of blood glucose and impaired carbohydrate, fat, and protein metabolisms. Chronic hyperglycemia in diabetics affects several major organs, including heart, blood vessels, nerves, eyes, and kidneys, thereby leading to diabetic complications, such as cardiac dysfunction, atherosclerosis, neuropathy, retinopathy, and nephropathy^2^,^3^. Peroxidation of membrane lipids induced by hyperglycemia contributes to the pathophysiology of cardiovascular disease and atherosclerotic foam cell formation in the arterial wall^4^. Diabetes, which is a silent disease, is the most prevalent disease in developed and underdeveloped countries, and its prevalence is increasing considerably. Diabetes has been considered as one of the fastest growing epidemic worldwide; the number of people with diabetes is estimated to increase from 381.8 million in 2013 to 591.9 million in 2035^5^.

The metabolic disorder type 2 diabetes or non-insulin-dependent diabetes mellitus is also associated with abnormal levels of lipids and lipoproteins, including cholesterol, low-density lipoprotein (LDL), very low-density lipoprotein (VLDL), high-density lipoprotein (HDL), triglyceride (TG), as well as leptin and adiponectin^6^. Intramyocellular lipid (IMCL), particularly long-chain fatty acyl-CoA and diacylglycerol, may possibly induce insulin resistance in the skeletal muscles through hexokinase activity inhibition or affect the insulin-signaling pathway. Accumulation of intramuscular lipid in sedentary people is associated with insulin resistance. Lipoprotein lipase (LPL) activity in skeletal muscles increases during heavy exercise activity, which is effective in reducing IMCL accumulation^7^. Leptin and Adiponectin, which are two major hormones produced by adipose tissue and associated with type 2 diabetes, normalize insulin action^8^. AMP-activated protein kinase (AMPK)-induced fat oxidation may mediate the effects of leptin and adiponectin, resulting in liver TG reduction and intramyocellular regulation or energy balance. Leptin stimulates insulin resistance whereas adiponectin elevates insulin sensitivity^9^.

Pharmacological studies have demonstrated the link between the presence of free radicals and degenerative disease. The crucial role of free radicals scavengers have been well explained^10^. At present, natural products or dietary phytochemicals have received significant interests as potential therapeutic agents to counter diabetes because of their valuable therapeutic properties, nontoxicity, and cost effectiveness^11^. Natural sources have strongly been suggested for curing diabetic patients instead of multiple insulin injections. However, anti-diabetic agents are required to regulate blood glucose levels in more serious conditions^12^,^13^. The potential therapeutic effects of the spice saffron have been documented over the last two decades. The body of evidence that saffron has possible anti-diabetic properties is growing. Saffron (Crocus sativus L.), belonging to the Iridaceae family, is commonly cultivated in Iran, Spain, India, and Greece. The phytochemical compounds of this plant include carotenoids (crocin and crocetin), glycoside (picrocrocin), and a volatile oil component (safranal). Crocin and crocetin, which are the most important carotenoids and major bioactive constituents of saffron, have a wide spectrum of biological activities^14^,^15^. Crocin, the main ingredient of saffron, has an anti-diabetic effect on male mice^16^. Crocetin reduces adiponectin expression suppression, insulin resistance caused by palmitate^17^, increase tumor necrosis factor alpha (TNF-α), and inhibit leptin expression increase in adipose tissue^18^.

Physical activity affects overall metabolism, especially of blood glucose and lipids. Therefore, it has a significant value in the treatment of type 2 diabetes, which is often managed by doing exercise modification. The interrelationships between physical activity and metabolic outcomes have been widely investigated^19^. For instance, lipid profiles and insulin sensitivity in type 2 diabetes have improved by doing aerobic exercise^20^. Moreover, exercise can also elevate Foxo1 expression, further reduce glycated hemoglobin (HbA1c), change blood lipid profiles, and further increase insulin sensitivity^19^. Physical activity has been proven to increase SIRT1 and Foxo1 levels by caloric restriction, leading to increased adiponectin levels^21^. Decreased fat mass with increased adiponectin levels because of physical activity has also been observed^22^. Exercise has been established to control or prevent diabetic disorder; however, the intensity, time, and mode of exercise programs for treatment have not been specified. Although scientific researchers provide many information on the biological properties of saffron but its acutely antidiabetic potential properties *in-vitro* and combined with exercise *in-vivo* have not yet been studied. Thus, the present study aims to establish the effectiveness of saffron on diabetic parameters *in-vitro* and combined with resistance exercise intervention *in-vivo* models.

## Research Design and Method

### Preparation of hydroalcoholic extract of saffron

The Saffron used in this study was obtained from Iranian premium Saffron provider (Saharkhiz Co.Mashhad-Iran) and identified by botanists in the herbarium with specimen number 829-537-06. To obtain and optimize the best quality technique for saffron compounds, different extractions method are performed^23^. Hydroalcoholic extract of saffron was prepared with 3.8 g of ground saffron added into 200 mL of 50% aqueous-alcoholic solution and soaked for 24 h. The prepared extract was filtered through a 0.2 mm filter and was concentrated by rotary evaporator to separate the hydroalcoholic solution and isolate the pure saffron extract. The resulting extract was concentrated under reduced pressure and stored at −20 °C until use. The 40 g of dried extract was dissolved in 1 mL of distilled water to arrive at the desired concentrations. The extraction method was repeated as much as needed.

#### Total phenolic content (TPC)

The total phenolic content was determined by the method described previously^24^. The procedure was carried out in triplicate and the total phenolic content of the samples were expressed in gallic acid equivalents (GAE) per gram of the sample (mg GAE/g dried weight).

#### Antioxidant activity

##### Ferric reducing power assay (FRAP)

The FRAP assay was used as a novel method for assessing “antioxidant power to measure the reduction of ferric to ferrous ion at low pH^25^. Positive controls were chosen to be ascorbic acid and the procedure was carried out in triplicate.

##### 1,1-diphenyl-2- picrylhydrazyl (DPPH) radical scavenging assay

The assay of DPPH radical scavenging activity was carried according to the method adopted by Blois *et al.*^26^. The experiment was done in triplicates and ascorbic acid was used as the positive controls. The percentage of inhibition was determined according to the following equation: % Inhibition = Control OD-Sample OD/Control OD × 100.

#### *In-vitro* cell line studies

##### Cell culture

Rat pancreatic beta cell line (RIN-5F) and rat L6 myoblast cell line (L6) were used in this study. RIN-5F and L6 cells were purchased from the American Type Culture Collection (ATCC, USA). RIN-5F (ATCC, CRL- 2058) cell were cultured in RPMI-1640 (Sigma–Aldrich, St. Louis, MO, USA) and L6 cells (ATCC, CRL-1458) were grown in Dulbecco’s Modified Eagle Medium (DMEM, Life Technologies, Inc., Rockville, MD, USA). The cells were supplemented with 10% fetal bovine serum (FBS, Sigma–Aldrich, St. Louis, MO, USA) and 1% antibiotics (100 IU/mL of penicillin and 100 μg/mL of streptomycin (iDNA, South America) and were maintained in a humidified 5% CO2 incubator at 37 °C. Cells were seeded in a flask at the required density per well and incubated for the desired time prior to the experiments.

##### Cell viability assay

The influence of saffron extract on RIN-5F and L6 cells was determined by the MTT [3-(4,5-dimethylthiazol-2-yl)-2,5-diphenyltetrazolium bromide] assay^27^. Cells were seeded in a 96-well plate at a density of 5 × 10^3 ^cells/well and incubated at 37 °C and 5% CO_2_. After 24 h, medium was replaced with a fresh medium. Different concentrations (0, 100, 250, 500, and 1000 μg/mL) of saffron extract were prepared and transferred to the cells in the 96-well plate and incubated for an additional 48 h. Then, 20 μL of MTT solution (5 mg/mL MTT bromide in PBS) was added and the mixture was incubated for 4 h at 37 °C. Then, the medium was removed and the MTT formazan crystals formed by the metabolically viable cells were dissolved in 100 μL of dimethyl sulfoxide. The absorbance was measured at 595 nm. The assay was performed in triplicate.

##### Insulin secretion by the cultured RIN-5F pancreatic cell

RIN-5F cells were seeded in 24-well plates at 2 × 10^5 ^cells/well and incubated at 37 °C and 5% CO_2_. After incubation for 24 h, the medium was removed from the wells, and the cells were washed twice with fresh medium containing low glucose (6.25 mM) or high glucose (12.5 mM)^28^. Afterwards, the cells were incubated at 37 °C for 3 h with the low glucose or high glucose medium, supplemented with 1% FBS and treated with low or high concentrations of saffron extract (200 and 400 μg/mL). Then, the aliquots in all wells were collected to determine the concentration of insulin in the media with the use of ELISA kit (Insulin ELISA kit, Ab100578, Abcam, Cambridge, UK) according to the manufacturer’s instructions. The insulin secretion levels at different concentrations of saffron were assessed by comparing with the control insulin secretion level. The 0 concentration of extract (untreated cell) was considered as the control. The experiment was conducted in triplicate and data are presented as mean ± SD.

##### Determination of glucose uptake by cultured L6 myotubes

L6 myoblasts cells were subcultured in 24-well plates at 5 × 10^4 ^cells/well and allowed to proliferate for 11 days to form myotubes in 0.4 mL of 10% FBS/DMEM. The medium was refreshed every 3 days. Then the 11-day-old myotubes were kept for 2 h in Krebs–Henseleit buffer (pH 7.4, 0.141 g/L of MgSO_4_, 0.16 g/L of KH_2_PO_4_, 0.35 g/L of KCl, 6.9 g/L of NaCl, 0.373 g/L of CaCl_2_-2H_2_O and 2.1 g/L of NaHCO_3_) containing 0.1% bovine serum albumin (BSA), 10 mM Hepes, and 2 mM sodium pyruvate (KHH buffer). The myotubes were thereafter cultured in KHH buffer containing glucose (normal: 11 mM; high glucose: 25 mM) without or with saffron extract (0, 200, and 400 μg/mL) for another 4 h. Glucose concentrations in the KHH buffer were determined with a glucose assay kit and a microplate reader (Appliskan, Thermo Fisher Scientific Inc., Waltham, MA, USA) at 508 nm, and the consumed glucose levels were derived from the differences in glucose concentrations between before and after culturing^29^.

### Protein extract and Western blot

**Figure 1:**
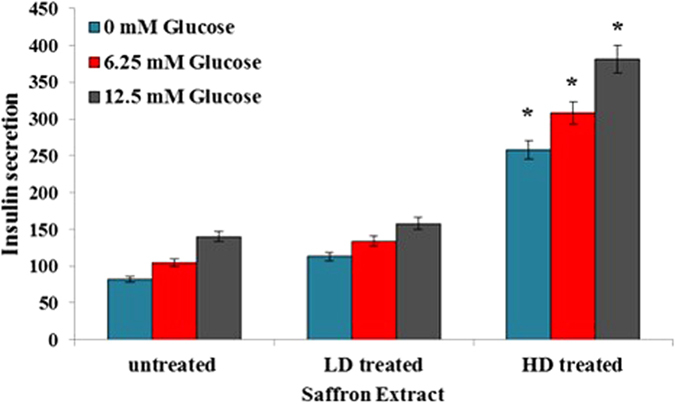
Effect of saffron extract on glucose stimulated insulin release in RIN5 cells. Data were expressed as mean ± SD for 6 replicates. LD; low dose of saffron-treated (200 μg/ml), HD; high dose of saffron-treated (400 μg/ml). *p value less than 0.05 considered as significant.

The expressions of GLUT2, GLUT4, and AMPKα were determined by Western blot. Briefly, total protein was extracted from RIN-5F and L6 myotube cells using lysis buffer (PRO-PREP Solution Kit, Intron, UK). The concentrations of the total extracted proteins were evaluated using a BSA standard kit (Thermo Scientific™ , US). With the use of 12% electrophoresis or sodium dodecyl sulphate polyacrylamide gel electrophoresis, the quantified proteins were separated to detect a specific protein and then transferred to a polyvinylidene fluoride membrane (BIORAD, UK) for immunoblot. The membranes were then incubated into specific primary and secondary antibodies (ABCAM, UK). The immunoreactive target protein bands were visualized using a colorimetric Opti-4CN TMS substrate kit. The captured target protein bands were analysed using Image J software (National Institutes of Health, USA). The ratio of each band over GAPDH was considered as the level of the target protein expression (Fig. 1).

### Experimental animals and ethical aspects

In this study, 70 male Sprague–Dawley rats 250 ± 15 g in weight, and 8 weeks of age were obtained from the animal center house of University Malaya and then transferred to the laboratory polycarbonate cages. They were reared at a temperature of 22 ± 2 °C and 55 ± 5% moisture under 12/12 h light/dark cycle. During the research process, the animals were fed with provided pellet food for animal breeding, reproduction, and stem cells. Water was allowed freely in special 500 mL bottles. All procedures involving animal experiments were approved and carried out in strict accordance with the United States Institute of Animal Research guidelines for the care and use of laboratory animals by the Animal Care and Use Committee (ACUC) University Malaya Institutional with ethics number: FIS/22/11/2011/FD(R).

#### *In-vivo* toxicity evaluation of saffron

In order to demonstrate the safety dosage of the plant extract, acute toxicity of the saffron was carried out in adult male and female rats. The experiment was conducted according to the guidelines of the Organization for Economic Co-operation and Development (OECD) No. 420^30^. Twenty male and female rats were assigned evenly into 2 groups and administered orally 200 and 400 mg/kg/day of the extract in a single dose, using intra-gastric tubes, or distilled water as vehicle. The animals were fasted overnight prior to the dosing (free access to water) and food was withheld for another 3 to 4 h after dosing. The animals were observed for 30 min and 2, 4, 8, 24 and 48 h following the administration to monitor any onset of clinical or toxicological symptoms. Any signs of toxicity, behavioral changes, and mortality were recorded over a period of 2 weeks. Sacrificed rats’ tissues (Liver & Kidney) were stained with hematoxylin and eosin,  × 20, and then high-resolution images were captured.

### Diabetes induction

After a week of acclimatizing the rats to the laboratory standard conditions, streptozotocin (STZ, Sigma–Aldrich, St. Louis, MO, USA) was injected intraperitoneally (55 mg/kg) to induce diabetes in rats^11^. Distilled citrate buffer was used to prepare the injection solution. Rats fasted 14 hours before injection. The animals in non-diabetic group were received citrate buffer injection as vehicle. A few days after STZ injection concentration 300 mg/dl were considered as diabetic. Blood samples were collected from the tail vein to measure the rats’ blood glucose using a glucometer (Bionime GM300). After one week of diabetes induction, the animals were transferred to conduct the treatment and experimental part.

### Experimental animal groups

For accurate comparison of study data, animal models were grouped into two: A) diabetic and B) non-diabetic groups. In diabetic group (A), animals were randomly distributed to five subgroups: 1) control (cn), 2) training (tn), 3) extract treatment (ext), 4) training + extract treatment (tn + ext) and 5) treated with metformin 100 mg/kg (met) and served as reference group. In non-diabetic group (B), animals were randomly distributed to four subgroups: 1) control (cn), 2) training (tn), 3) extract treatment (ext), and 4) training + extract treatment (tn + ext). Both non-diabetic and diabetic subgroups comprise 5 and 10 rats, respectively. Treated animals received intra-gastric hydroalcoholic extract of saffron at 40 mg/kg daily^31^.

### Resistance training protocol

After a week of acclimatization, the animals were first trained to climb the vertical ladder as the exercise for this study. The climbing familiarization sessions were conducted three to four replicates every day for a week without weights attached. Resistance exercise was performed from 4–8 p.m., and the extracted saffron treatment was administered at 10–11 a.m. with the use of a special gavage manufactured by Iran Supa Company. For 6 weeks, the training groups underwent 5 exercise sessions of climbing the ladder with attached weights weighing 30–100% of body weight per week. A specific ladder for rodents with a height of 1 m, with steps that are 4 cm apart and inclined at 85° angle was used. As warm-up for the training program, the animals climbed the ladder thrice without weights attached. The animals then performed their training twice with a weight attached to their tails, and then new weights were added. The training load consisted of four climbs with 50, 75, 90, and 100% of the previous maximum carrying load the animals were able to raise^32^.

### Blood sampling and determination of biochemical variables

Animals from the trained and sedentary treated groups were euthanized at the end of six weeks of resistance training and gavage procedure. The animals from the trained groups were decapitated 24 hours after the last training session. Animals were anaesthetized with ketamine/xylazine (80/8 mg/kg i.p.) and sacrificed by cervical dislocation. Scarifies were performed at the same period and all efforts were made to minimize suffering. Blood sample was collected through a heart puncture under similar conditions. In this study, insulin level was measured in micro-international units per milliliter (mcIU/mL). Insulin resistance was obtained using the previously validated homeostasis model assessment of insulin resistance (HOMA-IR); HOMA‐IR = fasting glucose (mmol/L) × fasting insulin (μU/mL)/22.5^33^. Serum glucose and total cholesterol were measured using an enzymatic glucose oxidase assay with a digital spectrophotometer (Spectronic, US), whereas fasting insulin level was measured using ELISA (enzyme-linked immunosorbent assay) kit. Adiponectin and leptin levels were measured by using ELISA Kit (Abcam-Cambridge, UK). LDL was calculated using the Friedewald equation^34^, and VLDL by VLDL = TG/5 formula. To measure HDL, HDL-C diagnosis kits were used following the photometric method.

## Statistical Analysis

All data are presented as mean ± standard deviation. Shapiro–Wilk test was conducted to determine the data are normally distributed. Leven’s test for homogeneity was conducted, and the data displayed a homogenous characteristic with p > 0.05. Analysis of variance (ANOVA) was performed using SPSS version 18 and applied to measures of central tendency and dispersion. Two-way ANOVA was used for comparing the effect of exercise and saffron extract and the combination of them on diabetic parameters. A Tukey post-hoc analysis was used to check for significant differences among the main effects of each dependent variable. Statistical significance was considered when p value was less than 0.05.

## Results

### Antioxidant properties of hydroalcoholic extract of saffron

As shown in Table 1, the total phenolic content of the samples are expressed in gallic acid equivalents (GAE) per gram of the sample (mg GAE/g dried weight). It indicates that the extract has the high TPC contents (9.02 ± 0.69 mg GAE/g DW) which is in line with previous study reported by^35^. Table 1 also shows the power of the hydroalcoholic extract of saffron in reducing ferric tripyridyl (Fe^3+^) to ferrous form (Fe^2+^). In fact, the reduction of the ferric tripyridyltriazine (Fe III TPTZ) complex to its ferrous form, which has an intense blue colour, can be monitored by measuring the change in the absorbance at 593 nm. The FRAP value obtained of the saffron extract (1.4 ± 0.21 mmol Fe^2+^/g of dried weight) was lower than that obtained of Ascorbic acid as positive control (5.1 ± 0.45 mmol Fe^2+^/g of dried weight). The results of correlations analysis calculated by SPSS, shows that there was a strong positive correlation between total phenolic and FRAP in the saffron extract (r = 0.998, p < 0.01). The anti-oxidant activity of the extracts was considered by using FRAP correlated significantly and positively with the total phenolic content (r = 0.998, p < 0.01). The results showed that the DPPH radical scavenging activity of the hydroalcoholic extract of saffron increases in a dose-dependent manner. The IC_50_ value obtained of the extract was 189 ± 7.6 μg/ml, though not as potent as ascorbic acid used as positive control (16 ± 5.84). IC_50_ of DPPH radical scavenging activity and total phenolic content were analysed by Pearson’s method. There was highly and negative correlation between total phenolic content in saffron extract with the IC_50_ of DPPH scavenging capacities (r = −0.996, p < 0.01). In the other words, negatively high correlation coefficient means that the higher amount of total phenolic content is related to lower IC_50_ of DPPH scavenging capacity. In fact, the highly and negative correlation between TPC with IC_50_ DPPH expressed better antioxidant activity. The results from this study have revealed the potential health benefit of saffron extract by scavenging free radicals.

### Cell proliferation assay

To assess the non-cytotoxic concentration of saffron, the viability of RIN-5F and L6 cells were evaluated at doses ranging between 0 and 1000 μg/mL, using MTT assay. Within the tested concentrations, saffron only showed negligible cytotoxicity at 800–900 μg/mL in both tested cell lines (data not shown), and concentrations up to 400 μg/mL of saffron were used in subsequent experiments.

### Insulin secretion by cultured RIN-5F pancreatic cells

As shown in Fig. 1, saffron extract markedly increased insulin secretion in a dose-dependent manner at glucose concentrations of 6.25 and 12.5 mM. A slight induction of insulin secretion was observed in the RIN-5F cells treated with saffron extract at a concentration of as low as 200 μg/mL. The induction level obtained at higher concentrations (400 μg/mL) was significant compared with that at a lower concentration (200 μg/mL) or not treated.

### Determination of glucose uptake by cultured L6 myotubes

**Figure 2:**
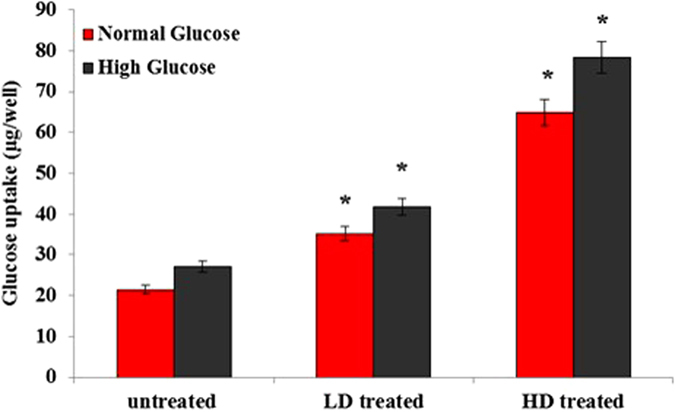
Effect of saffron extract on glucose uptake in L6 myotubes. Data were expressed as mean ± SD for 6 replicates. LD; low dose of saffron-treated (200 μg/ml), HD; high dose of saffron-treated (400 μg/ml). *p value less than 0.05 considered as significant.

As shown in Fig. 2, the glucose uptake of myotubes was considerably stimulated by the treatment of saffron in a concentration-dependent manner at concentrations of 200 and 400 μg/mL under normal glucose (11.1 mM) and high glucose (25 mM) conditions of the present study. This result suggests that saffron may act on the proteins associated with glucose uptake signaling pathways in muscle cells.

### GLUT2, GLUT4, and AMPKα protein expression quantification

**Figure 3:**
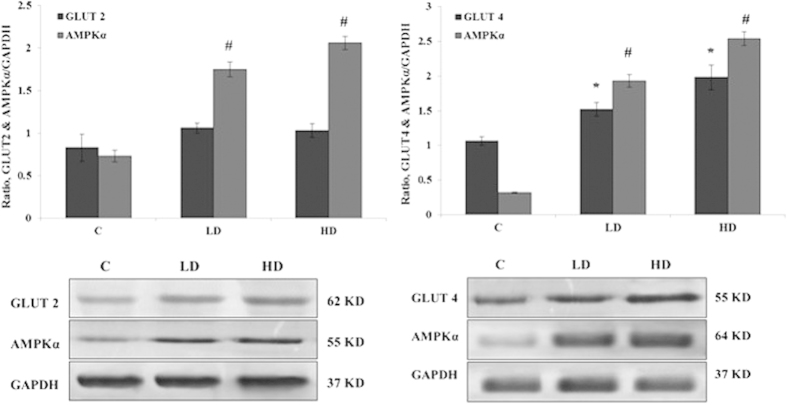
Western blot analysis for GLUT2, GLUT4, and AMPKα protein expression form of RIN-5F pancreatic cell and L6 myotubes cell with high or low dose of saffron treated compare to untreated. Data were expressed as mean ± SD. C; control, LD; low dose of saffron-treated (200 μg/ml), HD; high dose of saffron-treated (400 μg/ml). ^#&^*P value less than 0.05 considered as significant.

The protein blot expression analysis by Image J scanning (Fig. 3) indicated that GLUT2 expression approximately increased insignificantly by 0.2-fold in the pancreatic cells under high- or low-dose saffron treatment compared with the untreated RIN-5F pancreatic cell. GLUT4 expression increased significantly by 0.46 and 0.92-fold in the pancreatic cells under high or low-dose saffron treatment compare with the untreated L6 myotubes cell. AMPKα expression increased significantly by 1.02- and 1.33-fold, 1.61- and 2.22 fold in the RIN-5F pancreatic or L6 myotubes cell, respectively, under high- or low-dose saffron treatment compared with the untreated cells.

### Acute toxicity

**Figure 4:**
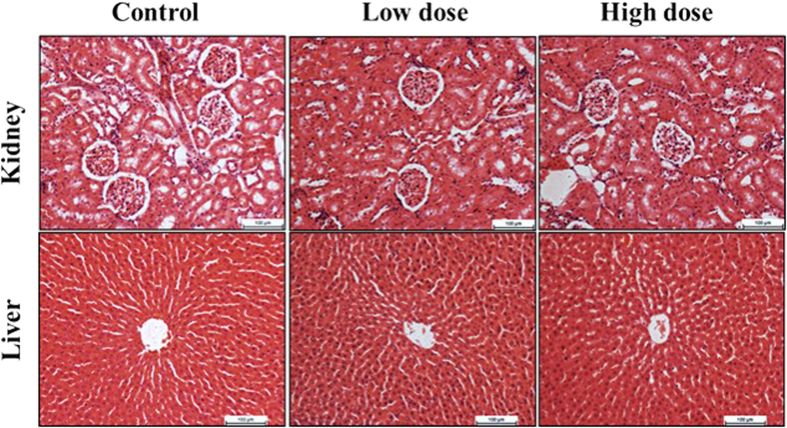
*In-vivo* toxicity evaluation of saffron on rat kidney and liver. Histological evaluation of rats’ kidney and liver tissues stained with H & E × 20 (Haematoxylin and Eosin). Data were collected from rats sacrificed at 7 days of saffron (low & high doses, 20 & 60 mg/kg/day) administration. The H & E investigation confirmed that there are no inflammation, abnormality, and organs lesions throughout the toxicity administration period.

Figure 4 presents the results of histological evaluation of liver and kidney in acute toxicity study. The result of the histopathologic examination confirmed that saffron is highly biocompatible for clinical applications. All of the animals were healthy without any sign of toxicity at the tested dose and during the experiment no mortality was recorded.

### Animal body weight in both diabetic and non-diabetic rats

The effects of Saffron or in combination with exercise on animal weight within 6 weeks are presented in Table 2. Rats in control diabetics groups were lost body mass as a result of the streptozotocin (STZ) throughout 6 weeks while tn, ext, and tn + ext groups had slightly increased of body weight at first 4 weeks then was significantly increased after 6 weeks in two groups of ext, and tn + ext. Further, over the six weeks of saffron treatment and exercise in non-diabetic groups no significant changes of body weight have observed during experimental study.

### Biochemical analysis

The Saffron and training effects on blood biochemical variables changes in A) diabetic and B) Non-diabetic groups are presented in Table 3.

#### Glucose

The blood glucose concentration levels in the tn, ext, tn + ext subgroups were significantly lower than that in the cn subgroups of both diabetic or non-diabetic groups (p < 0.05), and among the subgroups, the tn + ext subgroup exhibited the most reduction albeit not significant. In non-diabetic groups, the blood glucose concentration levels was significantly decreased in tn + ext group while there was no significant changes observed in tn and ext groups compared to control.

#### Insulin and insulin resistance

No significant differences exist among the diabetic and non-diabetic groups in terms of insulin concentration level. However, the insulin level insignificantly increased in the ext and tn + ext subgroups.

Insulin resistance levels were markedly decreased in the tn, ext, tn + ext subgroups compared with that in the cn diabetic subgroup, although no significant change was observed in non-diabetic groups.

#### *Glycated hemoglobin* (*HbA1c*)

The concentration level of HbA1c were significantly lower in the tn, ext, tn + ext subgroups compared with that in the cn diabetic subgroup, and among the subgroups, the tn + ext subgroup exhibited the most reduction albeit not significant. While, there was no statistically change in non-diabetic group.

#### Cholesterol and TG

The concentration levels of cholesterol was significantly lower in the tn, ext, tn + ext subgroups of both diabetic and non-diabetic groups, and among the subgroups, the tn + ext exhibited the most reduction albeit not significant. The level of TG concentration was markedly decreased in diabetic group compere to diabetic control while there was insignificant decrease in no-diabetic sub-groups in comparison with non-diabetic control.

#### HDL

The HDL concentration levels in all the groups (non-diabetic and diabetic) are not significantly different.

#### LDL and VLDL

The concentration levels of LDL and VLDL were significantly lower in both diabetic and non-diabetic groups compared with respected cn group. Meanwhile among the subgroups, the tn + ext subgroup exhibited the most reduction in both group albeit not significant.

#### Leptin

The leptin concentration levels in the both groups (non-diabetic and diabetic) are not significantly different.

#### Adiponectin

No significant differences were observed in the adiponectin concentration levels in both diabetic and non-diabetic groups.

## Discussion

The control and treatment of diabetes and its complications mainly depend on chemical or biochemical agents; however, a case of total recovery from diabetes has never been reported^36^,^37^. The data from the present study indicated that saffron extract increased insulin secretion by the RIN-5F cells. The stimulation of insulin secretion was significant in high-dose treatment. In addition, saffron could significantly stimulate glucose uptake in the L6 myoblast cells under hyperglycemic and normoglycemic conditions. Insulin secretion and glucose absorption were stimulated by saffron in a dose-dependent manner, which was more pronounced in high dose saffron treated cells. The result of the *in vivo* study showed that fasting glucose decreased in diabetic rats after six weeks of resistance exercise and/or saffron treatment which was in consist with *in-vitro* finding. With respect to the antioxidant results of saffron the possibility that it acts as redox-mediated mechanisms on insulin secretion and glucose uptake.

HbA1c reduction was observed after six weeks of resistance exercise and saffron treatment. Blood glucose level has a positive correlation with glycosylation of erythrocytes and HbA1C level. The beneficial effect of exercise on HbA1C reduction has been proved by multiple studies^7^,^18^,^38^,^39^. Moreover, fluctuations in body composition and weight loss can be the reasons for fasting blood glucose reduction, which is caused by metabolic marker improvement. Significant decreases in serum glucose, HbA1c^39^, and lipid profile in diabetic patients have been observed through exercise^40^,^41^. The data of the present study showed that the six-week consumption of the aqueous saffron extract reduced fasting glucose in diabetic rats. Furthermore, saffron supplementation combined with resistance training was more effective than either extract or resistance training (non-significant) alone in reducing fasting glucose. These findings are consistent with the results of previous studies on fasting blood glucose reduction with crocetin. Therefore, low levels of serum insulin with crocetin consumption show that crocetin can be considered as an effective treatment for treating insulin resistance or related diseases^17^,^42^,^43^. Medicinal properties of crocetin have been indicated to be associated with antioxidant activity as an important factor in the treated of many degenerative diseases^44^,^45^.

Hyperinsulinemia and enhancement of insulin resistance cause renal sodium retention, increased sympathetic tone, and vascular endothelium smooth muscle hypertrophy^46^. Six weeks of resistance training had no significant effects on insulin reduction in diabetic rats, which is contradictory to findings of several studies^40^,^47^,^48^. Such deviation can be ascribed to the subjects and diabetes induction process in rats. In some cases, streptozotocin has been shown to further damage pancreatic β-cells, whereas in other cases it is less destructive. However, this streptozotocin causes oxidative stress, apoptosis, and reduced production and release of insulin. The effect of exercise on insulin levels can vary. Therefore, whether exercise contributes to insulin or streptozotocin reduction cannot be determined definitively.

The findings of this study showed that six weeks of resistance training combined with saffron treatment resulted in insulin resistance reduction. These data results are consistent with previous findings^39^,^41^. The positive effects of resistance exercise on improved insulin resistance can be achieved through insulin receptor enhancement in muscle cells or an increase in the number of glucose transporter proteins in the skeletal muscle cells. GLUT4 from the glucose transporter proteins family is insulin-responsive and benefits glucose transportation through muscle contraction and insulin in the muscles. Hence, the increase in total muscle mass because of resistance exercise leads to an increased glucose uptake through insulin mediation^41^. Moreover, muscle contraction under metabolic stress conditions cause a rapid depletion of glycogen and change the binding of various proteins linked to glycogen. This function requires the considerable amount of muscle fibers to refill their carbohydrate reserves. Reconstruction of glycogen stores with changes in molecular structure and non-esterified fatty acids concentration reduction contribute to insulin sensitivity regulation in skeletal muscles after exercise training^49^. Therefore, an increase in lean body mass after resistance exercise may be an important mediator in the improvement of glycemic control. Probably because of resistance training, the muscle mass even without altering the intrinsic capacity of the muscle to respond to insulin improves glucose disposal^39^. The data of the *in-vitro* study through Western blot confirmed no significant increase in the GLUT2 and AMPKα expression in the RIN-5F cells, which is in line with *in-vivo* results.

Upregulation GLUT4 and AMPKα were observed in both low and high doses of saffron treated in L6 myoblast cell line. Saffron intensely stimulates phosphorylation of in both cell lines. AMPK performs many functions in fat or lipid synthesis of hepatic or skeletal muscle tissues, such as stimulating fatty acid oxidation, inhibiting cholesterol synthesis, and modulating insulin, adipocyte lipolysis, and muscle glucose uptake^43^. AMPK decrease production of glucose through various mediators e.g. by inhibiting gluconeogenic enzymes phosphoenolpyruvate carboxykinase (PEPCK) or translocating of GLUT-4 leads to stimulating glucose uptake. The anti-diabetic effects of natural products through increasing expression and promoting translocation of GLUT-4 via PI3K/AKT, CAP/Cb1/TC10 and AMPK pathways have been proven in previous studies. The data confirm previous findings on Saffron extracts in regards to strongly enhance glucose uptake^50^, as well as involvement of AMPK into this exact process in diabetic rats^51^. The results of current study suggest that the GLUT4/AMPK translocation and insulin resistance reduction may confirm a crosstalk between insulin metabolism, saffron compounds, and resistance training. Consequently, saffron may have a direct role in stimulating AMPK and GLUT4 via redox-mediated mechanisms for insulin sensitivity or glucose uptake in skeletal muscle cells.

No significant differences were observed in the adiponectin levels in diabetic rats after six weeks of resistance training and saffron consumption. Studies on the effect of saffron extract on adiponectin level are very limited. A study reported increased adiponectin levels in rats consuming 40 mg/kg of crocetin, which is consistent with the present data; such deviation may be caused by differences in saffron dosage^18^,^43^. Adiponectin enhancement by doing physical activity has also been reported^52^,^53^. The type of physical activity can be the cause for such inconsistency. Studies have shown that aerobic exercise has greater beneficial effect on adiponectin level compared with resistance training. Hypoadiponectinemia leads to greater insulin resistance and increased risk of type 2 diabetes^54^. Unlike most other adipokines, adiponectin levels in obese individuals are lower compared with those in lean people^55^,^56^,^57^. Nevertheless the fragmentation of adiponectin molecules is likely to be elevated in obese patients^58^. TNF-α decreases adipocyte-derived adiponectin expression *in-vitro*^59^ leading to insulin resistance^53^.

Furthermore, six weeks of resistance training and saffron consumption had a significant reduction in the total cholesterol of diabetic rats. Most researchers investigated the effect of saffron, with crocetin as the main efficient substance, on lipids and glycemic index^16^,^42^,^43^,^60^,^61^. Lower cholesterol after physical training can be due to changes in fat regulator enzymes and increased use of fats as fuel source during exercise. Crocin appears to indirectly inhibit the absorption of fat and cholesterol by preventing fat hydrolysis through the inhibition of pancreatic lipase. Crocin also increases the amount of fecal excretion of fat and cholesterol but had no effect on bile acids. A direct effect of crocin is the inhibition of lymphatic absorption of cholesterol^62^ as its roles as antioxidant agents have confirmed^63^. The present study showed saffron extract consumption had a significant reduction effect on TG, LDL, and VLDL. Studies have proved the positive influence of saffron on improved lipid profiles^62^,^64^. Crocin contained in saffron possibly causes an increased activity of lecithin–cholesterol acyltransferase (LCAT), which is involved in the regulation of blood lipid metabolism. LCAT also performs a vital function in the binding of free cholesterol to HDL^62^. Therefore, saffron and its constituents reduce lipid peroxidation^65^.

No significant differences in the HDL levels because of saffron consumption were observed in the present study. The findings are also inconsistent with the results of other studies. Asdaq and Inamdar reported an increase in HDL levels because of the use of saffron extract. Such an inconsistency could be partially related to different doses of saffron. In the present study, 40 mg/kg was the dose used; it might not have sufficient effect on the HDL compared with the doses used in previous research^64^. The present data also demonstrated no significant differences in the HDL levels after six weeks of resistance training, which is consistent with the findings of some previous studies^66^ but inconsistent with those of other studies^67^,^68^. However, this inconsistency might be related to the type, intensity, and duration of the exercise program. Jiméneza *et al.*^67^ conducted 8 weeks of resistance training, whereas other study set an endurance training of 90 min/day biking at 50% of peak^68^.

In the present study, a marked decrease was observed in the TG^69^, VLDL^68^,^70^ and LDL^54^ levels after six weeks of resistance training, which is supported by previous studies. Considering the positive changes in the lipid profile, physical activity protocols are recommended to increase lipolysis, decrease TG, and improve the ratio of oxidant to antioxidant, change LDL-C synthesis or plasma LDL-C elimination by tissue. Some investigations have suggested that changes in lipid profile by practice are possibly associated with a change in fat mass^71^. Physical activity causes an increase in LDL and LCAT and decrease in LTP (lipid transfer protein), resulting in HDL elevation and TG or VLDL reduction^72^. Greater LPL activity because of exercise performance and decrease in TG concentration create an increase in the LDL peak particle size^73^.

No significant changes were observed in the leptin levels in diabetic rats after six weeks of resistance training and saffron consumption. These findings are inconsistent with recent work^74^ but have been defended in several studies^75^,^76^. Leptin is an important factor involved in various processes, such as appetite regulation, metabolic function rate, reproduction, and immunity^76^. A higher leptin level is independently associated with increased risk of insulin resistance syndrome^60^ and is inversely related with insulin sensitivity^77^ and inverse relationship with insulin sensitivity^18^. Obese people generally have higher leptin concentrations. In these cases, leptin resistance could be induced by blood–brain barrier dysfunction, defects in leptin receptor signaling, or interference of neuropeptide Y^78^. Physical activities consequently reduce fat mass by creating a negative energy balance, which is mainly ascribed to the control of leptin. Despite the remarkable effect of physical activity, other factors may also be involved in leptin regulation. Subjects with lower fat mass have significantly lower response to plasma leptin caused by exercise compared with people with higher fat mass. In addition, the association between leptin and fat distribution possibly change with age^75^. Exercise training seems to improve leptin activity and sensitivity^78^.

## Conclusion

To sum up, the findings of the present study indicated that the consumption of the herbal plant saffron combined with resistance exercise is a strong therapeutic effective factor on diabetic parameters *in-vivo*. Our data were supported by *in-vitro* results. It was suggested that our two factors may improve diabetic parameters via GLUT4/AMPK pathway and may acts as redox-mediated mechanisms to regulate insulin or glucose. Thus, with the benefits of resistance training and concurrent saffron consumption on reducing health risk factors, diabetic patients are advised to exploit this combination of factors to control and manage their disease. Our study provides a key insight into diabetes, but significant evidences for the benefits of the combined factors have not been obtained. However, further investigations are warranted to identify the proper dose and role of specific compounds of saffron on diabetic parameters at the molecular level.

## Additional Information

**How to cite this article**: Dehghan, F. *et al.* Saffron with resistance exercise improves diabetic parameters through the GLUT4/AMPK pathway *in-vitro* and *in-vivo. Sci. Rep.* **6**, 25139; doi: 10.1038/srep25139 (2016).

## Figures and Tables

**Table 1:** Antioxidant activities of hydroalcoholic extract of saffron. Results are expressed as means ± SD of triplicate (n = 3).

Assays	TPC (mg gallic acid equivalent/g DW)	IC50 of DPPH Radical Scavenging Activity (μg/ml)	FRAP (mmol Fe^2+^/g DW)
Hydroalcoholic extract of saffron	9.02 ± 0.69	189 ± 7.6	1.4 ± 0.21

The IC_50_ values were calculated from linear regression analysis which indicates the effective concentration of samples used to reduce 50% of the reagent colour.

**Table 2:** Comparison of the effect of Saffron and Training on changes in both A) diabetic and B) Non-diabetic rat’s weight.

A) Diabetic groups	Control (cn)	Training (tn)	Extract treated (ext)	Training + extract treated (tn + ext)	Metformin (Met)
Day 1	251.2 ± 19.5	260.1 ± 16.4	258.2 ± 13.9	252.9 ± 11.8	255.3 ± 17.6
Week 2	243.7 ± 14.7	260.2 ± 18.7	259.9 ± 17.3	254.1 ± 15.4	258.3 ± 14.2
Week 4	231.9 ± 11.8	263.7 ± 19.9	265.6 ± 15.7	264.7 ± 19.5	261.3 ± 19.1
Week 6	223.1 ± 11.5	264.5 ± 20.2	271.7 ± 14.5*	272.8 ± 21.6*	270.9 ± 24.3
B) Non-diabetic groups					
Day 1	259.4 ± 13.3	255.7 ± 11.8	260.5 ± 16.1	258.1 ± 17.2	
Week 2	263.1 ± 12.5	251.4 ± 16.7	259.8 ± 14.7	250.6 ± 17.4	
Week 4	270.5 ± 12.4	244.3 ± 13.5	255.8 ± 19.2	241.2 ± 14.8	
Week 6	282.9 ± 18.5	244.5 ± 10.8	250.6 ± 18.3	232.1 ± 13.3	

**Table 3:** Comparison of the effect of Saffron and Training on changes of biochemical parameters in both A) diabetic and B) Non-diabetic groups.

A) Diabetic groups	Control (cn)	Training (tn)	Extract treated (ext)	Training + extract treated (tn + ext)	Metformin (Met)
Glucose mg/dl	392.20 ± 7.95	316.42 ± 9.04 *	305.28 ± 1.82*	292.28 ± 4.65*	162.9 ± 6.85*
Insulin mcIU/mL	6.04 ± 0.36	6.07 ± 0.36	6.15 ± 0.15	6.28 ± 0.30	5.98 ± 1.05
Insulin resistance μU/ml	6.25 ± 0.37	4.69 ± 0.31*	4.64 ± 0.13*	4.94 ± 0.21*	3.54 ± 0.62*
Glycated haemoglobin%	9.53 ± 0.35	7.40 ± 0.19*	7.02 ± 0.08*	6.96 ± 0.11*	6.32 ± 0.82*
Cholesterol mg/dl	122.85 ± 1.24	84.14 ± 1.63*	85.00 ± 0.57*	82.50 ± 1.49*	78.43 ± 2.02*
Triglyceride mg/dl	201.70 ± 4.48	152.28 ± 4.66*	143.00 ± 0.57*	138.33 ± 1.68*	119.37 ± 2.5*
HDL mmol/L	17.85 ± 0.73	19.42 ± 0.64	19.5 ± 0.15	18.93 ± 0.47	19.03 ± 1.29
LDL mg/dl	64.65 ± 1.08	37.25 ± 1.42*	36.90 ± 0.56*	34.50 ± 1.62*	40.84 ± 1.67
VLDL mg/dl	40.34 ± 0.89	30.45 ± 0.93*	28.60 ± 0.11*	27.67 ± 0.33*	29.62 ± 1.03*
Leptin ng/mL	3.28 ± 0.06	3.04 ± 0.29	2.78 ± 0.14	2.76 ± 0.08	2.13 ± 0.17*
Adiponectin ng/mL	6.31 ± 0.23	6.57 ± 0.70	6.50 ± 0.32	6.63 ± 0.23	7.91 ± 0.48*
B) Non-diabetic groups					
Glucose mg/dl	107.4 ± 2.33	102.8 ± 1.45	107.33 ± 1.81	97.00 ± 1.62*	
Insulin mcIU/mL	15.30 ± 0.15	15.29 ± 0.17	15.47 ± 0.15	15.27 ± 0.13	
Insulin resistance μU/ml	4.06 ± 0.12	3.88 ± 0.09	4.10 ± 0.09	3.66 ± 0.08	
Glycated haemoglobin%	1.98 ± 0.08	1.89 ± 0.07	1.86 ± 0.07	1.95 ± 0.07	
Cholesterol mg/dl	70.85 ± 1.47	66.02 ± 1.69	68.33 ± 0.98	59.45 ± 0.93	
Triglyceride mg/dl	75.14 ± 2.62	61.30 ± 1.57	65.88 ± 1.94	55.18 ± 1.31	
HDL mmol/L	31.87 ± 1.88	33.70 ± 1.26	33.22 ± 1.53	32.72 ± 1.36	
LDL mg/dl	24.22 ± 2.54	18.70 ± 2.50*	18.93 ± 2.02*	14.69 ± 1.97*	
VLDL mg/dl	15.02 ± 0.52	12.26 ± 0.31*	11.77 ± 0.38*	11.03 ± 0.26*	
Leptin ng/mL	3.05 ± 0.08	2.91 ± 0.05	2.76 ± 0.09	2.77 ± 0.02	
Adiponectin ng/mL	6.23 ± 0.28	6.12 ± 0.06	5.39 ± 0.22	5.23 ± 0.25	
